# Integrity of Multiple Memory Systems in Individuals With Untreated Obstructive Sleep Apnea

**DOI:** 10.3389/fnins.2020.00580

**Published:** 2020-06-24

**Authors:** Melinda L. Jackson, Genevieve Rayner, Sarah Wilson, Rachel Schembri, Lucy Sommers, Fergal J. O’Donoghue, Graeme D. Jackson, Chris Tailby

**Affiliations:** ^1^Turner Institute for Brain and Mental Health, School of Psychological Sciences, Monash University, Melbourne, VIC, Australia; ^2^School of Health and Biomedical Sciences, RMIT University, Melbourne, VIC, Australia; ^3^Institute for Breathing and Sleep, Austin Health, Melbourne, VIC, Australia; ^4^The Florey Institute of Neuroscience and Mental Health, Melbourne, VIC, Australia; ^5^Melbourne School of Psychological Sciences, University of Melbourne, Melbourne, VIC, Australia; ^6^Clinical Epidemiology and Biostatistics Unit, Murdoch Children’s Research Institute, Melbourne, VIC, Australia; ^7^Faculty of Medicine, Dentistry and Health Sciences, University of Melbourne, Melbourne, VIC, Australia

**Keywords:** autobiographical memory network, sleep, working memory, fMRI, hypoxia

## Abstract

Obstructive sleep apnea (OSA) is associated with working- and autobiographical-memory impairments, and high rates of mood disorder. This study aimed to examine (i) behavioral responses and (ii) neural activation patterns elicited by autobiographical and working memory tasks in moderate-severe untreated OSA patients and healthy controls, and (iii) whether variability in autobiographical and working memory activation are associated with task performance, OSA severity and psychological symptomatology (depression, anxiety). In order to control for the potential confounding effect of elevated rates of clinical depression in OSA, we excluded individuals with a current psychiatric condition. Seventeen untreated OSA participants and 16 healthy controls were comparable with regards to both activation and behavioral performance. OSA was associated with worse subclinical mood symptoms and poorer personal semantic memory. Higher levels of nocturnal hypoxia were associated with increased activation in the occipital cortex and right cerebellum during the working memory task in OSA participants, however, no significant relationships between activation and task performance or depressive/anxiety symptomatology were observed. The neurocognitive substrates supporting autobiographical recall of recent events and working memory in younger, recently diagnosed individuals with OSA appear to be indistinguishable from healthy age-matched individuals. These findings point to the importance of early diagnosis and treatment of OSA in order to preserve cognitive function.

## Introduction

Obstructive Sleep Apnea (OSA) is a common sleep disorder, caused by collapse of the pharyngeal airway during sleep producing intermittent hypoxia, frequent arousals from sleep, and fragmented sleep patterns (Young et al., 2002). It is estimated to affect ~4% of middle aged adults (Young et al., 1993), rising to up to 60% of the elderly (Ancoli-Israel et al., 1991). OSA is associated with significant daytime consequences, including excessive sleepiness, depressive symptoms, and cognitive deficits (Jackson et al., 2011a), with an increased risk of cognitive decline and Alzheimer’s disease (Bubu et al., 2020). Approximately half of all patients with OSA have some form of memory impairment, and are at risk of premature age-related memory decline (Yaffe et al., 2011). OSA is also associated with changes in brain morphology (Morrell et al., 2010) and brain network activity, both of which are associated with cognitive impairments (Canessa et al., 2011).

Impairments of both the working memory and autobiographical memory systems have been reported in OSA. Working memory (WM) refers to the cognitive system that temporarily maintains and stores information while simultaneously permitting processing and manipulation of that information (Baddeley, 2003). WM impairments are frequently (Naëgelé et al., 2006; Cosentino et al., 2008), though not always (Twigg et al., 2010), observed in OSA. Autobiographical memory (AM) is the record of our experiences throughout life. We recently reported significant impairments in the ability of OSA patients to recall specific autobiographical events (Lee et al., 2016), and to retrieve personal semantic information (Delhikar et al., 2019) compared to healthy controls. Such impairments are linked to significant reductions in productivity, mood, and quality of life (Sumner et al., 2010).

Previous functional imaging studies have reported altered activity within the WM system in OSA, with associated poorer performance on WM tasks in some (Thomas et al., 2005; Ayalon et al., 2009), but not all (Castronovo et al., 2009), studies. A large body of literature shows that WM tasks recruit a frontoparietal network of brain areas (Owen et al., 2005). The frontoparietal WM system is generally considered antagonistic to the default mode-like network of areas that is consistently activated by AM tasks (Rekkas and Constable, 2005; Tailby et al., 2017), comprising midline regions (medial prefrontal, medial temporal, and retrosplenial/posterior cingulate cortex), lateral cortical regions (ventrolateral prefrontal, anterolateral temporal, and temporoparietal cortex), and the cerebellum (Spreng and Grady, 2009). Despite neuroanatomical overlap between AM activation and gray matter volume changes reported in OSA (Morrell et al., 2010), no studies have directly examined the functional correlates of AM in OSA.

The current study aimed to determine if (i) there are differences in behavioral measures of cognitive function (autobiographical and working memory), and in subclinical psychological (depressive or anxiety symptoms) function, between untreated OSA patients and age-matched controls; (ii) there are differences in activation patterns elicited by autobiographical and working memory tasks in OSA patients compared to age-matched controls, and (iii) changes in autobiographical and working memory activation are associated with alterations in task performance, measures of OSA severity, and subclinical symptomatology (depressive or anxiety symptoms).

## Materials and Methods

### Participants

The sample consisted of 17 participants with untreated, recently diagnosed OSA (within the last 6 months) and 16 healthy controls. Inclusion criteria for the OSA group were: aged 18–65 years; confirmed diagnosis of OSA as determined by clinical diagnostic polysomnography (apnea hypopnea index > 10); English fluency; and right handed. Potential participants (OSA and control) were excluded if they had: a current active psychiatric disorder (including depression or anxiety) or medical condition including epilepsy, recent stroke, myocardial infarction (in last 6 months); head injury with loss of consciousness > 15 min; learning disability; alcohol or drug dependence; were pregnant or possibly pregnant; shiftworkers; or any contraindications to having an MR scan. While four participants had a SCID diagnosed episodes of depression in the past, no participants were excluded for an active/current diagnosis of depression at the time of their participation. Healthy control participants were recruited through (1) advertising flyers posted at Austin Health, and (2) healthy family members recruited from an fMRI study of autobiographic memory in epilepsy (Tailby et al., 2017). Inclusion and exclusion criteria were the same as per the OSA group, except they were to have no OSA or other sleep disorder, as determined by self-report. The study was approved by the Austin Health Human Research Ethics Committee, and all methods were performed in accordance with relevant guidelines and regulations.

### Procedure

After providing written informed consent, each participant completed questionnaires, a 2 h neuropsychological test battery and a 1 h fMRI scan at the Melbourne Brain Centre, Austin Health. Participants had the option of undertaking the scan and neuropsychological test battery on separate days. Participants were provided with a $50 gift voucher for their involvement in the study.

### Materials

#### Screening Questionnaires

Participants completed the Hospital Anxiety and Depression Scale (HADS) (Zigmond and Snaith, 1983); the Structured Clinical Interview for DSM-IV Axis I Disorders (First et al., 1994); the Test of Premorbid Function (TOPF); and the Epworth Sleepiness Scale (ESS) (Johns, 1991).

A *Modified Vividness of Visual Imagery Questionnaire* (VVIQ) (Amedi et al., 2005) was used to ensure equivalence between groups in their ability to produce mental images, an important element of autobiographical recollection, allowing us to discount poor ability to generate internal visual imagery as contributing to any group differences found in brain activation.

The *Autobiographical Memory Interview* (AMI) (Kopelman et al., 1990) is a semi-structured interview that assesses explicit episodic and semantic memories sampled from three specific time periods: childhood, early adulthood, and recent life. This provides a cross-section of memories from across the lifespan, as well as total number of personal semantic and episodic memories recalled. The Personal Semantic Schedule requires participants to recollect personally relevant facts across the three time-points (e.g., former addresses; maximum score = 63), with scores ≤ 47 associated with an amnestic syndrome and 48–49 a probable amnestic syndrome. The Autobiographical Incident Schedule asks participants to recall three episodes from each time period (e.g., a wedding ceremony). Episodic memories are scored from 0 to 3 (maximum = 27) based on their richness in detail and how precisely the incident is located in place and time, with total scores ≤ 12 associated with an amnestic syndrome, and 13–15 a probable amnestic syndrome. Inter-rater reliability is *r* = 0.83–0.86, with good sensitivity to organic disease.

#### In Scanner Tasks

Participants completed an in-scanner *autobiographical memory (AM) recollection* task and a *n-back* (2-back) working memory (WM) task. Details of the AM paradigm (Tailby et al., 2017) and WM paradigm (Trevis et al., 2017) have been reported previously. Briefly, AM was probed via presentation of questions related to the participants’ standardized scripted interaction with research staff during the study (e.g., “where were you when you received the study reminder phone call?”). Participants were asked to mentally reflect or put themselves back in those situations to the best of their ability. Only the two recent (< 24 h) memory conditions described previously (Tailby et al., 2017) were utilized. The visual n-back paradigm (Trevis et al., 2017) was performed between the first and second runs of the memory conditions. This probed the WM system by having participants view sequentially presented letters and responding (via button press) whenever the current letter matched that shown two previously. The WM task also contained a condition requiring responses whenever the current letter was an “X,” and a condition requiring responses to all letters.

#### Post-scanner Task

To check that participants had been able to retrieve an AM while completing the in-scanner AM task we administered a questionnaire (adapted from Buchanan et al., 2006) to explore the internal details of the AMs recollected. Participants were asked to rate each memory on a 7-point scale for the items: depth of recollection, ease of recollection; pleasantness; intensity; significance; novelty; vividness; and frequency of rehearsal. Scores for each item were averaged across the two runs.

### MRI Acquisition, Processing, and Analysis

MRI was performed on a 3T Siemens Skyra scanner (Erlangen, Germany) using a 20-channel head coil. Functional images were acquired using a whole-brain gradient-echo single shot echo-planar imaging sequence (echo time 30 ms, repetition time 3,000 ms, field-of-view 72 × 72 voxels in-plane, 44 slices, voxel size 3 mm isotropic).

Data were preprocessed using SPM12. Functional images were slice time corrected, motion corrected, coregistered to the T1 image, warped to Montreal Neurological Institute (MNI) space [using DARTEL (Ashburner, 2007); specifically, the deformation from own space to the DARTEL template space was combined with the deformation from the DARTEL template space to MNI space, with the latter derived by running SPM’s segment routine on the mean of the DARTEL space T1 images], and smoothed (8 mm FWHM). In order to define a group wise brain mask for voxel-wise statistical testing (see next section), within brain masks for each participant were generated using the brain extraction tool from FSL (Smith, 2002) and their intersection calculated.

Images were analyzed via the general linear model, using SPM12. The multisession design matrix for the AM paradigm included, for each session, a regressor modeling the memory recollection periods—obtained by convolving the SPM canonical Hemodynamic Response Function (HRF) with a 48s boxcar spanning the recollection blocks – and the motion parameters estimated during preprocessing. The design matrix for the WM paradigm included a regressor modeling the 2-back blocks (HRF convolved with 22.5 s boxcar), a regressor modeling the detect “X” blocks (HRF convolved with 22.5 s boxcar), a regressor modeling the task-cue periods (HRF convolved with 1.5s boxcar) and the motion parameters estimated during preprocessing (see Trevis et al., 2017 for details). For the AM activation analysis the contrast of interest was the beta estimate for the recollection regressor (summed across sessions); for the WM analysis it was the difference between the beta estimates for the 2-back and detect “X” regressors.

### Statistical Analyses

One participant from the control group had missing data on the AMI, leaving 15 in the final analysis. Due to a technical fault, one participant’s data from the OSA group was missing for the n-back task, leaving 16 in the final analysis for this task. Independent samples *t*-tests and χ^2^-tests were conducted to compare demographics, AMI, n-back and questionnaire data between groups. For the AM and WM scanning paradigms we used *t*-tests (spm-t images) to contrast activation estimates in controls and OSA patients. The two resulting spm-t images (one for AM and one for WM) were feature thresholded at *p* < 0.001 followed by family wise cluster correction at *p* < 0.025 (0.05/2). Estimated smoothness (and RESEL counts) for the AM and WM analyses were [11.6, 11.1, 11.1] mm (955.4 RESELs) and [12.0, 11.5, 11.4] mm (870.7 RESELs), respectively. To investigate the relationship between BOLD activation, and sleep related variables, mood and performance outcomes in OSA patients, we regressed oxygen desaturation index (ODI), apnea hypopnea index (AHI), lowest oxygen desaturation (SaO_2_ nadir), ESS, HADS depression, HADS anxiety, and in-scanner WM scores on activation estimates in the AM and WM paradigms. The 13 resulting exploratory spm-t images were feature thresholded at *p* < 0.001, followed by family wise cluster correction at *p* < 0.003846 (0.05/13). Mean estimated smoothness and RESEL counts (± SEMs) across these regression analyses were [12.8, 12.2, 12.1] mm (± [0.2, 0.2, 0.2] mm) and 736.3 (± 39.1) RESELs, respectively.

## Results

### Higher Mood Symptoms and Poorer Personal Semantic Memory in OSA Patients

Table 1 displays the demographic, sleep, and performance variables for each group. The average AHI for the OSA group was in the moderate to severe range. While the groups overlapped in age, there was a significantly greater proportion of males in the OSA group. OSA participants reported significantly higher depressive [*t*_(30)_ = 7.59, *p* < 0.001] and anxiety [*t*_(30)_ = 3.55, *p* = 0.001] symptomatology than controls, but were mostly in the mild symptom range [i.e., < 11; Table 1; (by design, no participant had a clinical diagnosis of depression or anxiety disorder)]. There was no difference in estimated IQ between groups. Results from the behavioral tasks and vividness questionnaires are shown in Table 2. OSA participants recalled significantly fewer personal semantic memories on the Autobiographical Memory Interview (AMI) overall [*t*_(30)_ = −2.4, *p* = 0.02], however, there was no significant between groups difference in the number of autobiographical/episodic events recalled (*p* = 0.95). There were no significant between group differences in performance on the in-scanner working memory (*p*’s > 0.05), nor in VVIQ scores (*p* = 0.62). For the post-scanner task, OSA participants reported significantly lower vividness of memory recall compared to controls [*t*_(30)_ = −3.5, *p* = 0.002].

**TABLE 1:** Demographics, mood, and sleep variables for the OSA and healthy control groups.

	OSA	Controls
	*N* = 17	*N* = 16
Age	43.6 ± 13.7 (20–62)	35.25 ± 14.4 (23–61)
Gender (% males)**	76.5%	31.3%
BMI	31.9 ± 6.3 (21.8–40.0)	–
AHI events/h	49.5 ± 21.8 (25.4–95.9)	2.4 ± 1.1 (1.0–3.7)
Estimated IQ (TOPF)	103.6 ± 10.0 (87–120)	105.6 ± 14.0 (79–128)
Years of education*	14.1 ± 3.4	17.4 ± 3.5
HADS – depression***	6.8 ± 2.5 (2–10)	1.4 ± 1.4 (0–5)
HADS – anxiety**	7.4 ± 2.6 (2–12)	4.3 ± 2.2 (1–10)
ESS	10.4 ± 4.2 (3–19)	5.0 ± 0.8 (4–6)

Mean ± SD (range); *p < 0.05; **p < 0.01; ***p < 0.001. For the control group, N = 15 for HADS and N = 4 for AHI and ESS. For the OSA group, N = 9 for Years of education. AHI, apnea hypopnea index; BMI, body mass index; ESS, Epworth Sleepiness Scale; ToPF, test of premorbid functioning; HADS, Hospital Anxiety and Depression Scale.

**TABLE 2:** Behavioral outcomes for the OSA and healthy control groups.

	OSA	Controls	Cohen’s *d*
	*N* = 17	*N* = 16	
VVIQ	57.82 ± 9.98	59.59 ± 10.12	0.18
AMI personal semantic*	55.9 ± 3.1	58.6 ± 3.1	0.87
AMI autobiographical incidents	21.9 ± 3.6	21.9 ± 2.7	0.02
2-back hits	0.88 ± 0.09	0.90 ± 0.09	0.22
2-back hits RT (ms)	487.5 ± 89.4	465.6 ± 94.6	0.23
2-back false positives	0.05 ± 0.04	0.03 ± 0.03	0.57
2-back false positives RT (ms)	668.1 ± 196.7	587.7 ± 122.1	0.49

Mean ± SD (range); *p < 0.05. For the control group, N = 15 for AMI. For the OSA group, N = 16 for the n-back task. AMI, Autobiographical Memory Interview; RT, reaction times; VVIQ, Vividness of Visual Imagery Questionnaire.

**FIGURE 1:**
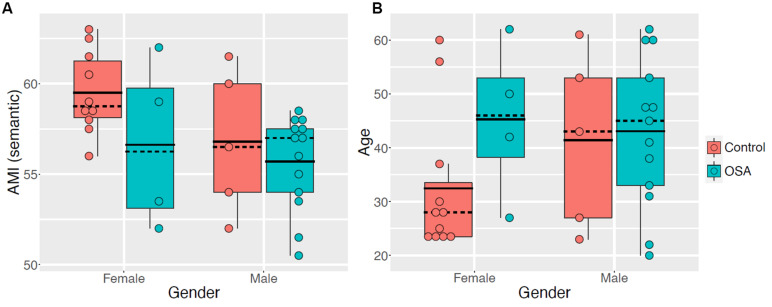
Boxplots, with overlaid dot plots, of **(A)** AMI semantic scores and **(B)** age, stratified by sex (Male, Female) and group (Control, OSA).

Given the disparity in sex between groups and the broad age range, further examination of AMI semantic scores by sex were examined (Figure 1A). There is a trend for AMI semantic scores to be worse, on average, in OSA for both males and females, suggesting OSA affects personal semantics regardless of sex. We also explored the potential impact of age on the AMI results, due to the broad range of ages across each group (Figure 1B). The boxplot shows the relatively even spread of ages across the OSA group for both sexes, and for the male controls. Further, the mean AMI semantic score in female controls is comparable regardless of whether those aged under 25 are included (μ = 59.5, *SD* = 2.3) or excluded (μ = 59.3, *SD* = 2.8), suggesting the subset of young females among the controls is not biasing our result.

### Comparable Activation Patterns in OSA and Controls

**FIGURE 2:**
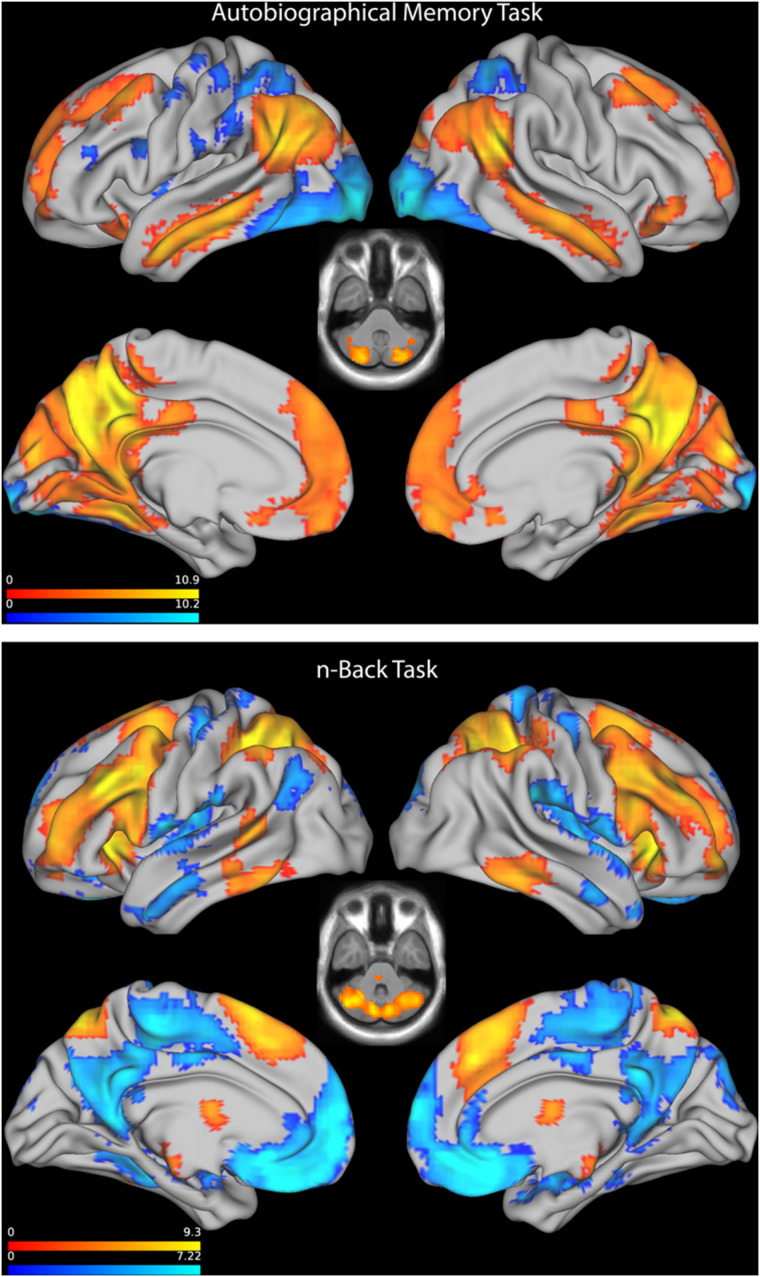
**Top:** Statistical parametric map showing activation, combined across controls, and OSA patients, on the Autobiographical Memory Task. Axial plane through cerebellum shown at *z* = −39. **Bottom:** Statistical parametric map showing activation, combined across controls and OSA patients, on the n-Back Task. Axial plane through cerebellum shown at *z* = −34.5. SPMs are feature thresholded at *p* < 0.001, followed by family wise cluster thresholding at *p* < 0.05.

As expected, the AM paradigm produced strong activation in medial and lateral parietal, posterior cingulate, retrosplenial, middle temporal, parahippocampal, medial prefrontal, and dorsolateral prefrontal cortex (Figure 2, top). There were no significant differences in the activation between OSA and controls.

The WM task resulted in strong activation in dorsolateral frontoparietal areas, anterior insula, superomedial frontal cortex and thalamus, along with deactivation in default mode areas, across both groups (Figure 2, bottom). Again, there were no significant differences in activation between OSA and controls.

### Measures of Hypoxia Are Associated With Increased Working Memory Activation

**FIGURE 3:**
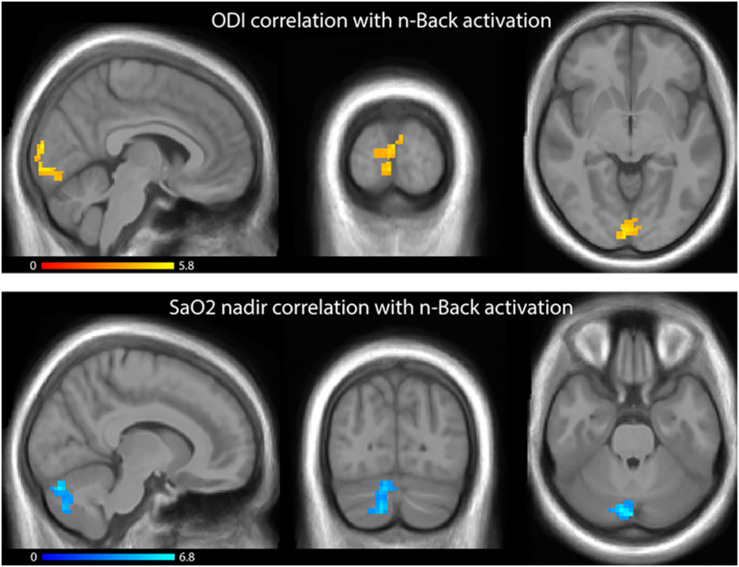
**Top:** Statistical parametric map showing, in OSA patients, regression of ODI on beta values from the n-Back task regressors; increasing ODI values were associated with increased activation. **Bottom:** Statistical parametric map showing, in OSA patients, regression of SaO_2_ nadir on beta values from the n-Back task regressors; decreasing SaO_2_ values were associated with increased activation. SPMs are feature thresholded at *p* < 0.001, followed by family wise cluster thresholding at *p* < 0.00625.

Regressions of ODI and SaO_2_ nadir on WM activation revealed significant clusters in occipital cortex and right cerebellum, respectively (Figure 3), with worsening hypoxia (higher ODI and lower SaO_2_) associated with greater activation in these clusters. No significant clusters were observed when ODI or SaO_2_ nadir were regressed on AM activations, or when ESS or AHI were regressed against activation on the AM or the WM tasks. There were no significant associations observed between activation and either in-scanner WM task performance, out-of-scanner AMI performance or HADS depressive/anxiety symptoms.

## Discussion

In this sample of moderate-severe untreated OSA patients, we found significantly worse personal semantic memory, along with significantly worse subclinical depressive and anxiety symptoms relative to controls. Autobiographical recall and working memory abilities were comparable between the groups. Our imaging analyses revealed no significant differences between OSA and controls in activation for autobiographical recall of recent events or working memory. However, higher levels of nocturnal hypoxia were associated with increased activation during the working memory task in the occipital cortex and right cerebellum in OSA participants. This is the first study to our knowledge to examine activation patterns elicited by autobiographical recall of recent events in OSA patients. The activation profiles of both groups during the AM and the WM tasks are consistent with previous studies in healthy controls (Svoboda et al., 2006; Tailby et al., 2017; Trevis et al., 2017).

In a larger independent OSA cohort, we previously observed that personal semantic memory, but not autobiographical recall of events, is impaired in OSA (Delhikar et al., 2019). Similar deficits have also been reported in early Alzheimer’s disease (Leyhe et al., 2009), raising the question of whether this may be a useful early marker of cognitive decline in individuals with OSA. Despite explicitly excluding cases with clinical psychiatric diagnoses, our sample of OSA patients also endorsed higher subclinical depressive and anxiety symptoms, which are common in this population (Jackson et al., 2011b, 2019b). In the present study we observed no between group difference in the activation patterns on the AM task, nor any association with depressive symptoms. The absence of between groups differences may reflect the emphasis of the in-scanner AM task on episodic autobiographical recall of recent events (on which the groups did not differ), as opposed to semantic aspects of AM. The AM task, as utilized here, cannot be separated into episodic- and semantic-related activation, given the comingled recruitment of both types of AM expected to occur during task execution. Specifically, during the in-scanner AM task participants were asked to recollect recent events such as traveling to and arriving at the research facility, whereas on the AMI, personal semantic memory is indexed via the ability to recall longer-term “factual” information such as the names of teachers and friends, and addresses from the school years. This points to a fractionated profile of autobiographical recollection in OSA that could be examined more closely in future work, using appropriate paradigm designs (Renoult et al., 2012).

One possible reason for the lack of association between activation patterns and depression/anxiety symptom is our exclusion of cases with diagnoses of psychiatric conditions. Given that rates of clinical depression are elevated in OSA (Jackson et al., 2019a), and the independent impact of depression on autobiographical memory processes, we explicitly chose to exclude participants with psychiatric diagnoses, so that any between group differences could be more confidently attributed to OSA *per se*. This may, however, have resulted in a restricted range of depressive symptomatology, limiting sensitivity to detecting associations with depressive symptoms. This raises the question of the impact of comorbid conditions (e.g., obesity, cardiovascular disease, depression), which are extremely common in OSA, on cognitive functioning and brain structure and function in this group. Disentangling the impact of comorbidities on brain health in OSA will require carefully designed studies that control for these potential confounds. Clinically, this also highlights the importance of screening for and treating mood disturbance in OSA patients, which could be contributing to cognitive impairments and daytime symptoms, as well as potentially impacting OSA treatment adherence.

The results of the WM task contrast with one previous study (Castronovo et al., 2009) that found increased activation of the working memory network in severe OSA patients compared to controls in the presence of intact performance, although these authors used less stringent control for multiple comparisons. More recently, no difference on 2-back performance or activation patterns between OSA patients and controls were observed, whereas OSA patients had poorer performance and greater deactivation in the cuneus region during the more demanding 3-back condition (Prilipko et al., 2011). It is possible that the 2-back version of the task employed in the current study was too simple, and a more complex task is required to expose working memory impairment in OSA. Of note, these previous studies have also assessed relatively young, moderate to severe OSA patients like those in the current study. Studies comparing older and younger adults with OSA have found that the combined effects of older age and OSA produce significant neural and behavioral deficits greater than either aspect alone (Ayalon et al., 2010). The current study adds to this literature by demonstrating that younger (<65 years), highly educated and relatively healthy patients with moderate-severe OSA have intact activation within neural systems supporting autobiographical recall of recent events and WM compared to age-matched controls. As already noted, we also excluded individuals with clinical depression. These features of our sample may endow them with greater cognitive reserve, enabling greater compensation, and concealing deficits early in the disease process (Cabeza et al., 2018). Similar processes have been posited to operate in the early stages of degenerative disease, with life experiences, leisure activities, and education and occupational attainment having a protective effect (Stern, 2012). Whether activation would have differed between groups during personal semantic memory recall is not known, however, since the paradigm used here cannot be used to parse autobiographical recollection in this manner.

Within the OSA group, greater hypoxia was associated with increased activation in cerebellum and occipital cortex during the WM task (Figure 2). Others (e.g., Prilipko et al., 2011) have also reported associations between duration of hypoxia and less deactivation within the default mode network during WM performance. This contrasts with previous reports of reduced cerebellar (but increased parietal) activation associated with increasing disease severity, as measured by AHI, during a 2-back working memory tasks, although this was only in a small sample of 9 OSA patients (Archbold et al., 2009). Reduced bilateral cerebellar gray matter volumes, maximal on the left, have been reported previously in OSA patients by our group (Morrell et al., 2010) and in a recent meta-analysis (Huang et al., 2019), suggesting a possible region of vulnerability associated with OSA. Cerebellar functions extend beyond motor control, and also involve cognitive and emotional processes (Schmahmann, 2004). Cerebellar activation and deactivation is commonly reported in imaging studies of WM, including n-back tasks (Cohen et al., 1997). Increased cerebellar activation in those with more severe hypoxia may reflect a compensatory mechanism, increasing attentional control in order to maintain performance during cognitive challenge.

One of the limitations of OSA studies is that the length of time that an individual has had OSA is unknown and difficult to determine, along with the severity of their OSA over the course of the condition. Future studies would benefit from understanding the role of cognitive reserve capabilities in OSA, given the association between OSA and cognitive decline (Yaffe et al., 2011), and that often cognition is not “normalized” after effective treatment (Jackson et al., 2018). Other limitations of this study are the small sample size, which may result in Type II errors, range of ages, sex differences, and obesity in the OSA group. While there are known sex differences in activation patterns of AM, this is specific to semantic autobiographical memory, whereas no sex differences were found in activation patterns for episodic AM previously (Compère et al., 2016). In addition, sex difference in activation patterns during AM recollection do not translate to sex differences at the behavioral level (Piefke et al., 2005). Future studies would benefit from a sample large enough to examine both sex and age differences in AM. We also did not assess all 16 controls for OSA with polysomnography, and there is a possibility – albeit small – that some of this group may have had undiagnosed OSA. Regardless of these limitations, the insights from studies such as ours are important for understanding the neural substrates of cognitive functioning in OSA patients, particularly in light of the growing body of evidence that untreated OSA puts an individual at a greater risk of later-life cognitive decline (Yaffe et al., 2011; Bubu et al., 2020).

In conclusion, this study found impaired personal semantic memory recall in recently diagnosed, non-depressed OSA patients compared to age-matched controls. Importantly, we found no difference in autobiographical recall of recent events, WM performance, or activation during AM and WM tasks. Those individuals who experience greater nocturnal hypoxia showed increased occipital and cerebellar activation during the WM task, potentially reflecting a compensatory mechanism. These preliminary findings may suggest that in younger individuals with OSA, in whom the neurocognitive substrates supporting personal autobiographical recollection and working memory appear intact, early treatment may be important as a means of minimizing progressive age- and OSA-related cognitive dysfunction.

## Data Availability Statement

The datasets generated for this study are available on request to the corresponding author.

## Ethics Statement

The studies involving human participants were reviewed and approved by Austin Health Human Research Ethics Committee. The patients/participants provided their written informed consent to participate in this study.

## Author Contributions

MJ, SW, GR, GJ, and FO’D contributed to the conception, design of the study, and oversaw all aspects. LS, RS, GR, and CT contributed to the acquisition and analysis of data. CT provided imaging analyses and figure generation. All authors contributed to editing the manuscript.

## Conflict of Interest

The authors declare that the research was conducted in the absence of any commercial or financial relationships that could be construed as a potential conflict of interest.
